# Germination response to water availability in populations of *Festuca pallescens* along a Patagonian rainfall gradient based on hydrotime model parameters

**DOI:** 10.1038/s41598-021-89901-1

**Published:** 2021-05-20

**Authors:** Aldana S. López, D. R. López, M. V. Arana, D. Batlla, P. Marchelli

**Affiliations:** 1 IFAB (Instituto de Investigaciones Forestales y Agropecuarias Bariloche; INTA-CONICET), Modesta Victoria 4450, 8400 Bariloche, Río Negro Argentina; 2 INTA, EF Villa Dolores, Córdoba, Argentina; 3 IFEVA/Catedra de Cerealicultura, Facultad de Agronomía, Universidad de Buenos Aires, Av. San Martín 4453, C1417DSE Buenos Aires, Argentina

**Keywords:** Physiology, Plant sciences, Ecology

## Abstract

Sensitivity to water availability is a key physiological trait for grassland species located in arid and semiarid environments, where successful germination is closely related to rainfall dynamics. *Festuca pallescens* inhabits diverse environments along a steep precipitation gradient in North Patagonia, thus offering a suitable model for the study of germination behavior in response to water availability. By analyzing germination in nine populations using a hydrotime model approach, we aimed to find within-species variation. Seed population behavior was analyzed under different hydric conditions using hydrotime model parameters (hydrotime, mean base water potential and its standard deviation). We estimated the mean base water potential for *F. pallescens* (ψb_(50)_ = − 2.79 ± 0.45 MPa), which did not differ significantly between populations. However, the hydrotime parameter (θ_H_) varied markedly, suggesting physiological adaptation to local environments. Higher values of θ_H_ were found in populations located at the extremes of the distribution gradient, indicating that germination may be prevented or delayed in conditions that are suboptimal for the species. Since the variation in hydrotime model parameters did not follow a cline, micro-environmental cues may have the greatest influence on the physiological behavior of the species, rather than the macroscale rainfall gradient.

## Introduction

Arid, semi-arid and sub humid regions are vulnerable to environmental fluctuations and anthropic overuse^1^. Many of these ecosystems are grasslands in which the spatio-temporal variability of temperature, precipitation, soil water availability and/or evapotranspiration favors the formation of environmental gradients^2,3^. The potential of grassland species to adapt to these environmental gradients under current climatic change partially depends on their ability to produce viable seeds and cope with environmental stress during germination^4^. In several grassland species germination is erratic or episodic^5,6^, and seeds may show different physical traits and adaptive germination mechanisms to facilitate their establishment in dry conditions^7^.

Germination is modulated by both genetic and environmental factors^8^, such that water availability and suitable temperatures are of overriding importance^9^. Dormancy also plays an important role in controlling the timing of germination^10–12^. Since germination begins with imbibition by the seed, water availability is a paramount limiting factor^10,13^. In the field, the water available for germination depends on the position of the seeds in the soil profile, the soil characteristics, and the weather conditions after a rainfall event^9^. Soil water content fluctuates widely due to rainfall and evapotranspiration cycles, and is particularly variable near the surface where germination and establishment are more likely to take place. Seeds may hydrate rapidly after rainfall in these upper soil layers, but they will also dry out more quickly. Therefore, successful plant establishment might be jeopardized in areas with low rainfall, xeric soils, and high temperatures during spring and summer.

Field studies evaluating germination and long-term probability of emergence are limited by their need for many years of field data^6^. Alternatively, threshold models are useful for describing and quantifying seed response to diverse conditions^14^. These models constitute a helpful tool for the evaluation of germination in the short-term, since they focus on knowledge of the seed parameters associated with temperature, water availability, light, after-ripening, chilling, etc^11,14–16^. In particular, germination patterns that respond to the water potential (ψ) of the seed environment can be described by the hydrotime model^17^. Hydrotime analysis provides indices of seed germination response (i.e., speed and uniformity) to water stress by estimating seed population parameters such as base water potential (ψb) and the hydrotime constant (θ_H_). The former parameter is the minimum water potential required for germination to occur^18,19^, while the latter defines the MPa units above ψb that seeds should accumulate in order to germinate^17^. This model has been widely used in weeds^12,20^, herbs^21^, forage shrubs^22^ and horticultural crops^23–25^. However, few studies have focused on the native forage species of South American rangelands^6,26^.

The Patagonian steppe constitutes the largest southernmost dryland ecosystem of South America^27^. Strong west winds and a sharp rainfall gradient determine the vegetation distribution in this region^28,29^. Rainfall ranges from 3000 mm in the west, near the Andes Mountains, to less than 150 mm towards the east, in the Patagonian steppe, and has a strong effect on the vegetation^30,31^. A key species in Patagonian grasslands is *Festuca pallescens* (St. Ives), a perennial, long-lived cool season bunchgrass that reproduces mainly through seeds^31–33^. Semi-arid *F. pallescens* rangelands cover an area of approximately 5200 km^2^ in Patagonia^33^. Due to the palatability of this species, these lands have been overgrazed since the beginning of the twentieth century, producing a decline in grasslands that provides clear evidence of vegetation deterioration^34–36^. Furthermore, over the past 5 years a drastic reduction in the abundance of *F. pallescens* has been observed, attributable to changes in climatic conditions^36^.

Populations of *F. pallescens* showed marked differences in seed sensitivity to temperature. Using a thermal time approach^37^, we found that the minimum temperature for germination (Tb = − 0.47 ± 0.19 °C) did not differ among populations. However, the thermal time required for germination varied significantly among populations from different environments, being higher in xeric than in humid environments (213.7 °Cd vs 144.24 °Cd, respectively). A correlation between seed physiological traits and temperature was thus identified. Nevertheless, to understand the germination requirements of the species along its complete longitudinal range in North Patagonia further analysis is required in terms of hydric requirements for germination. Therefore, the present study aims to expand current knowledge of the physiological germination requirements of the species by exploring seed responsiveness to changes in water availability during germination. This is evaluated in the same set of populations and related to the environmental variables that characterize the habitats of the populations. Given the steep rainfall gradient, water availability may limit germination towards the drier east, imposing strong selection pressure on *Festuca pallescens* populations. Accordingly, we hypothesise that seed responsiveness to water availability during germination will differ between populations inhabiting humid environments and those growing in xeric environments. These differences may be associated with local hydric environmental characteristics, reflecting the local adaptation of populations to their home environments, which may preclude germination at each site until the optimal temporal moment. We estimated the hydrotime parameters and tested for statistical differences between populations. In addition, we estimated correlations between the hydrotime parameters and environmental variables.

## Methods

### Plant material and germination tests

Seeds were collected from nine populations of *F. pallescens* at five sampling sites. The sites were situated along a west–east decreasing rainfall gradient that covers about 500 km in North Patagonia. Sampling was performed in January of 2015 at the moment of seed dispersal (Table 1). At each location, two populations from different altitudes (high steppe and wet-meadow) were sampled, except on Somuncura Plateau (SA) where only one altitude was sampled. In this region, low-altitude environmental conditions are too arid for *F. pallescens* to occur^35^. The sampling sites covered three ecological regions that include different communities of vegetation^35,38^: *Andean region* (populations PHA and PHB), *Pre-Andean region* (populations SRA and SRB) and *Hills and Plateaus* (populations PA, PB, JA, JB and SA) (see Table 1). We collected a pool of seeds from between 30 and 50 plants in each population, and stored them in darkness at 10 °C for four months until the experiments were carried out. We used a tetrazolium test^39^ to evaluate the average viability of the seed batch in each seed population. The seed weight of each population was determined with four replicates of 100 seeds each, to calculate thousand-seed weight. Permission to collect *Festuca pallescens* was given by the following institutions: Secretaría de Ambiente de Rio Negro (Argentina), Secretaría de Desarrollo territorial y ambiente de Neuquén, and Áreas protegidas y recursos faunísticos de la provincia de Neuquén, Argentina. Both the experimental research and field studies on *Festuca pallescens*, including the collection of plant material, complied with the relevant institutional, national, and international guidelines and legislation.

**Table 1:** Characteristics of the sampled *Festuca pallescens* populations: geographic location, thousand-seed weight and floristic physiognomic type^35^. Soil symbols correspond to those in Fig. 2.

Population	Sample site	Mean precipitation (mm)	South latitude	West longitude	Altitude (m)	Thousand-seed weight (gr)	Floristic physiognomic type	Type of soil	Soil symbols
PHA	Península Huemul Ranch, Neuquén, Argentina	831	40° 57′	71° 25′	1220	2.04	Shrub-grass steppe	Fairly deep, volcanic	
PHB			41° 1′	71° 20′	845	1.16	Shrub-grass steppe	Deep, sandy	
SRA	San Ramon Ranch, Río Negro, Argentina	584	41° 1′	71° 4′	1139	1.14	Grass steppe	Fairly deep sandy loam	
SRB			41° 7′	71° 1′	890	0.94	Meadow	Deep sandy loam with high proportion of Organic Soil Matter (OSM)	
PA	Pilcaniyeu Experimental Field, Río Negro, Argentina	264	41° 4′	70° 34′	1260	1.38	Shrub-grass steppe	Fairly deep sandy loam	
PB			41° 3′	70° 30′	970	1.25	Meadow	Deep loamy sand, alkaline with high proportion of OSM	
JA	Ingeniero Jacobacci, Río Negro, Argentina	170	41° 55′	69° 12′	1400	0.76	Grass steppe	Fairly deep sandy loam	
JB			41° 46′	69° 21′	970	1.51	Salty Meadow	Deep silty loam, saline-alkaline with high proportion of OSM	
SA	Somuncura plateau, Río Negro, Argentina	150	41° 25′	66° 58′	1430	1.1	Meadow	Fairly deep sandy loam	

Germination tests were performed during the first year after harvesting. Seeds were surface-sterilized with sodium hypochlorite (1%) for 1.5 min and rinsed with VITAVAX-FLO fungicide (Luján Agrícola, Argentina). Four water potentials (ψ = 0, − 0.8, − 1, − 1.6 MPa) were generated by the dissolution of different concentrations of polyethylene glycol (PEG 6000) in deionized water^40^ at 15.5 °C; all PEG solutions were verified with an osmometer. We used the average viability of each seed batch to calculate the number of seeds to place in each dish (four replicates per population per water potential) in order to achieve at least forty viable seeds per dish. Seeds were placed in 9-cm-diameter Petri dishes containing a single filter paper moistened with 10 ml of the different PEG isotonic solutions. The petri dishes were sealed with plastic film to prevent moisture loss. In order to keep the water potential of the incubation medium constant during germination experiments, the filter papers and PEG solutions were changed every 48 h. The experiment was run in a germination chamber, in darkness, at a constant temperature of 15.5 °C, since this temperature was estimated as optimal for the species^37^. A seed was considered germinated on protrusion of the radicle. Seed germination at the different water potentials was monitored every two days for 80 days for the time-course germination curves. The germination of each population was considered complete when no further germination was recorded for at least five consecutive days. At the end of the experiment, non-germinated seeds were tested for viability by tetrazolium; only seeds with red-stained embryos were considered for the estimation of germination percentage. In some cases, seeds were evidently rotten and were considered dead. Experiments for SA at − 1.6 MPa failed, so they were not included in further analyses.

### Estimation of hydrotime parameters

The time-course cumulative germination curves obtained for seeds incubated under the four water potentials were used to estimate the time required to complete the germination of the subpopulation percentiles (5–90%). The accumulated germination data was adjusted using the Gompertz equation, and the germination rate was calculated in the same way as for the thermal time model^37^. The germination rate of a given fraction g of the population (GR_(g)_) is linearly related to the water potential^18,19^; therefore, for each fraction of the population, a linear regression of GR_(g)_ was fitted to define the ψb_(g)_ of each fraction as the interception of the ψ axis when GR_(g)_ = 0. The hydrotime model generally assumes a constant value of θ_H_ and a variable value of ψb for different fractions of the seed population^14,16,17^. Most linear regressions for each population were parallel to each other (Supplementary Fig. 1 in Electronic Supplementary Material), so θ_H_ was considered a constant value defined as the inverse of the slope of the regressions (1/θ_H_), and ψb varied for different fractions of the seed population. Therefore, the hydrotime model parameters were calculated from the Gummerson model^18^ with the ψb normally distributed among the populations with a mean (ψb_50_) and standard deviation (σ_ψb_), according to (1, 2):

1 $$\theta_{H} { } = { }\left[ {{ }\psi - { }\psi {\text{b}}\left( {\text{g}} \right)} \right]/{\text{tg ,}}$$

where θ_H_ is the hydrotime constant (MPa d) for the population, ψ is the actual seed water potential (MPa), ψb (g) is the base or threshold water potential (MPa) defined for a specific germination fraction g, and tg is the time required for radicle protrusion of percentage g^17^. Equation (1) was reformulated to illustrate the relationship between ψ and the germination rate (GRg = 1/tg, the inverse of the emergence time of the radicle) of fraction g of the seed population:

2 $${\text{GRg }} = { }1/{\text{tg }} = { }\left[ {{ }\psi - { }\psi {\text{b}}\left( {\text{g}} \right)} \right]{ }/\theta_{H} { }{\text{.}}$$

All calculations and comparisons, including cumulative germination curves and linear regressions, were performed using GraphPad Prism demo version 5.00 for Windows (GraphPad Software, San Diego California USA, www.graphpad.com). The values of ψb (g), θ_H_ and σ_ψb_ were calculated for each replicate using a non-linear least squares curve fitting method (Premium Solver Platform 7.0, Frontline systems, Incline Village, NV, USA^20^). An ANOVA with heterogeneity of variances was performed in InfoStat (Di Rienzo et al. 2020) to assess differences in parameters ψb (g), θ_H_ and σ_ψb_ between populations. High θ_H_ values indicate that a long time is needed for germination (MPa d; i.e., slow germination) whereas low (i.e., more negative) values of ψb mean that seeds will germinate across a wider range of water potentials. Finally, high values of σ_ψb(50)_ indicate high temporal germination variation within the population. We also analyzed the relation between seed weight and hydrotime parameters with GraphPad Prism demo version 5.00 program for Windows, GraphPad Software, San Diego California USA, (www.graphpad.com).

### Relations between hydrotime parameters and environmental variables

We evaluated correlations between the hydrotime parameters of each population and the local environmental variables at the sampling sites. The environmental variables were obtained from Worldclim (https://www.worldclim.org/data/index.html). Relations between ψb, θ_H_ and σ_ψb_ and the following precipitation variables were assessed: annual precipitation, precipitation seasonality (i.e., coefficient of variation in the range of annual precipitation), autumn (April–June) and spring (September–November) precipitation (Supplementary Table A1). In addition, associations were analyzed between ψb, θ_H_ and σ_ψb_ and the altitude, longitude and latitude of each population. Linear regressions were performed with GraphPad Prism demo version 5.00 for Windows, GraphPad Software, San Diego California USA (www.graphpad.com), and the significance was then evaluated with the R^2^ adjustment of Eq. (3).

3 $$R^{2} = { }1 - [\sum \left( {y_{obs} - y_{pred } } \right)^{2} / \left. {\sum (y_{obs } - \overline{y}_{obs} )^{2} } \right].$$

In order to determine which variables show more environmental variability, we first carried out a Principal Component Analysis (PCA) and then a clustering analysis using Infostat^41^. The PCA describes the relation between the environmental variables and the parameters of the hydrotime model, while the hierarchical clustering method groups populations according to their similarities in the hydrotime model parameters and the environmental and physiognomic characteristics of each sampled site. Cluster analyses allow the implementation of different processes to group a set of environmental variables. Type of soil and floristic physiognomic type were used as classification variables. The hierarchical clustering algorithms were the Average linkage method and the Euclidean distance. Results are shown in a dendrogram—a tree diagram in two dimensions where the branches in the tree represent the clusters.

## Results

### Cumulative germination curves and estimation of the hydrotime parameters

**Figure 1:**
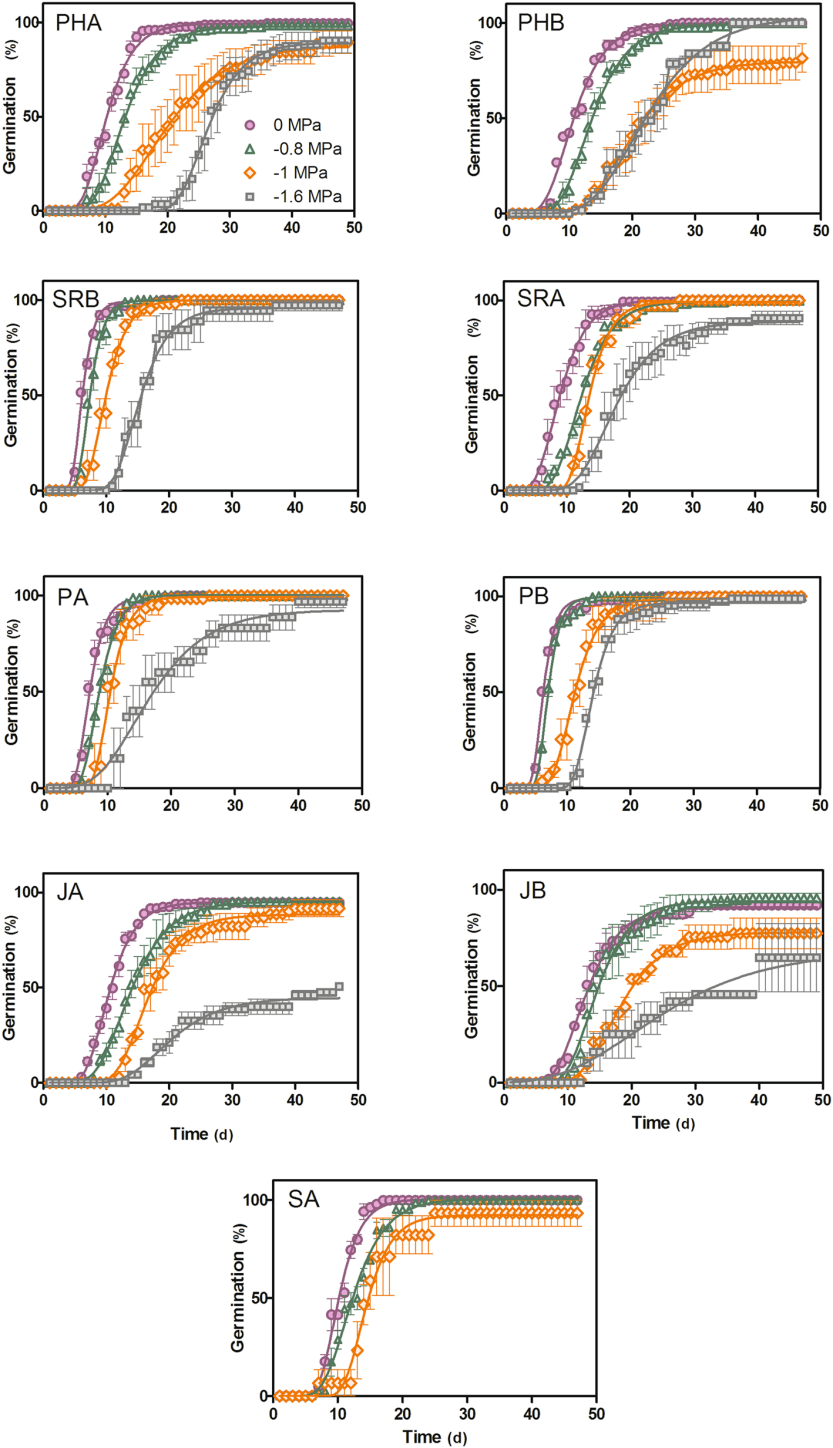
Cumulative germination curves for the nine populations of *Festuca pallescens*. The germination time-course curves at 0, − 0.8, − 1 and − 1.6 MPa were calculated with non-linear adjustment to the Gompertz equation. The SA population did not germinate at − 1.6 MPa (i, r). The vertical bars represent the standard error of the mean (SEM). The coloured symbols correspond to the working water potentials: Violet circle (0 MPa), green triangle (− 0.8 MPa), orange square (− 1 MPa) and grey square (− 1.6 MPa).

The time‐course cumulative germination curves show that all populations reached full germination at ψ = 0 and ψ = − 0.8 MPa except for JA, where germination reached 80–90%. The germination percentage was lower in more negative water potentials, as expected, and did not reach full germination (Fig. 1). When the seeds were exposed to ψ = − 1 and ψ = − 1.6 MPa, only SRB and PB completed germination (100%). Between 70 and 80% germination was recorded for the remaining populations, except for the three easternmost ones, two of which (JA and JB) germinated less than 50% at ψ = − 1.6 MPa. The experimental and theoretical germination curves for all populations and water potentials presented a very good fit to the model (R^2^ > 0.85) (Fig. 1, Table 2).

**Table 2:** Estimated parameters of the hydrotime model: ψb_(50)_, mean base water potential (MPa); θ_H_, hydrotime (MPa d); σ_ψb_, standard deviation of base water potential for each population; and mean R^2^ of the observed and predicted germination. Different letters indicate significant differences (Tukey, p < 0.05).

Population	Ψb_(50)_	θ_H_	σ_ψb_	R^2^
PHA	− 2.59 ± 0.11a	23.41 ± 0.34ab	0.57 ± 0.12a	0.86
PHB	− 3.14 ± 0.46a	30.93 ± 5.41b	0.91 ± 0.11a	0.85
SRA	− 3.04 ± 0.26a	22.01 ± 2.88ab	0.83 ± 0.09a	0.86
SRB	− 2.84 ± 0.82a	14.13 ± 3.47a	0.60 ± 0.36a	0.95
PA	− 2.91 ± 0.61a	17.49 ± 3.71ab	0.64 ± 0.17a	0.93
PB	− 2.69 ± 0.04a	13.63 ± 0.35a	0.58 ± 0.14a	0.93
JA	− 2.13 ± 0.09a	18.55 ± 0.67ab	0.54 ± 0.09a	0.92
JB	− 2.66 ± 0.67a	30.53 ± 10.3b	0.79 ± 0.39a	0.86
SA	− 3.12 ± 1a	27.76 ± 9.99ab	0.74 ± 0.27a	0.9

The hydrotime model was based on a variable ψb and a constant θ_H_ in the different fractions of the population (Supplementary Fig. 1 in Electronic Supplementary Material), which were adjusted using Eqs. (1) and (2) to calculate the values of ψb_(50)_, θ_H_ and σ_ψb_ for each population. The ψb_(50)_ values varied among the populations sampled along the precipitation gradient, ranging between − 2.13 (JA) and − 3.14 MPa (PHB). However, these differences were not significant (F = 1.31; p = 0.28, Table 2), and no increasing or decreasing pattern was detected in the ψb_(50)_ related to the rainfall gradient. The σ_ψb_ values did not show significant differences between populations (F = 1.36, p = 0.26) (Table 2). On the other hand, θ_H_ varied significantly between populations, PHB registering the highest value (30.93 MPa d) and PB the lowest (13.63 MPa d) (F = 5.43; p = 0.0006; Table 2). In addition, although seed weight differed among populations (F = 27.7; p < 0.001), it did not correlate with either hydrotime parameter (ψb_(50)_: r^2^ = 0.07; p = 0.47; θ_H_: r^2^ = 0.01; p = 0.75).

### Relationship between the parameters of the hydrotime model and the environmental variables of each population

Neither the hydrotime (θ_H_) nor the mean base water potential (ψb_(50)_) showed a significant correlation with the environmental or geographic variables; however, a slight gradient-associated pattern was observed between θ_H_ and precipitation seasonality (r^2^ = 0.4) and autumn precipitation (r^2^ = 0.4). The highest values for precipitation seasonality (indicating greater variability in precipitation) coincided with populations exhibiting low values of θ_H_ (PA, PB and SRB), while the lowest values for precipitation seasonality (low precipitation variability) coincided with populations with high values of θ_H_ (e.g., in the easternmost population [SA]). In addition, the highest θ_H_ values were found in the populations located at the extremes of the rainfall gradient (PHB in the west and JB in the east) with contrasting levels of precipitation in autumn, PHB having greater rainfall than JB (Supplementary Fig. 1 in Electronic Supplementary Material).

**Figure 2:**
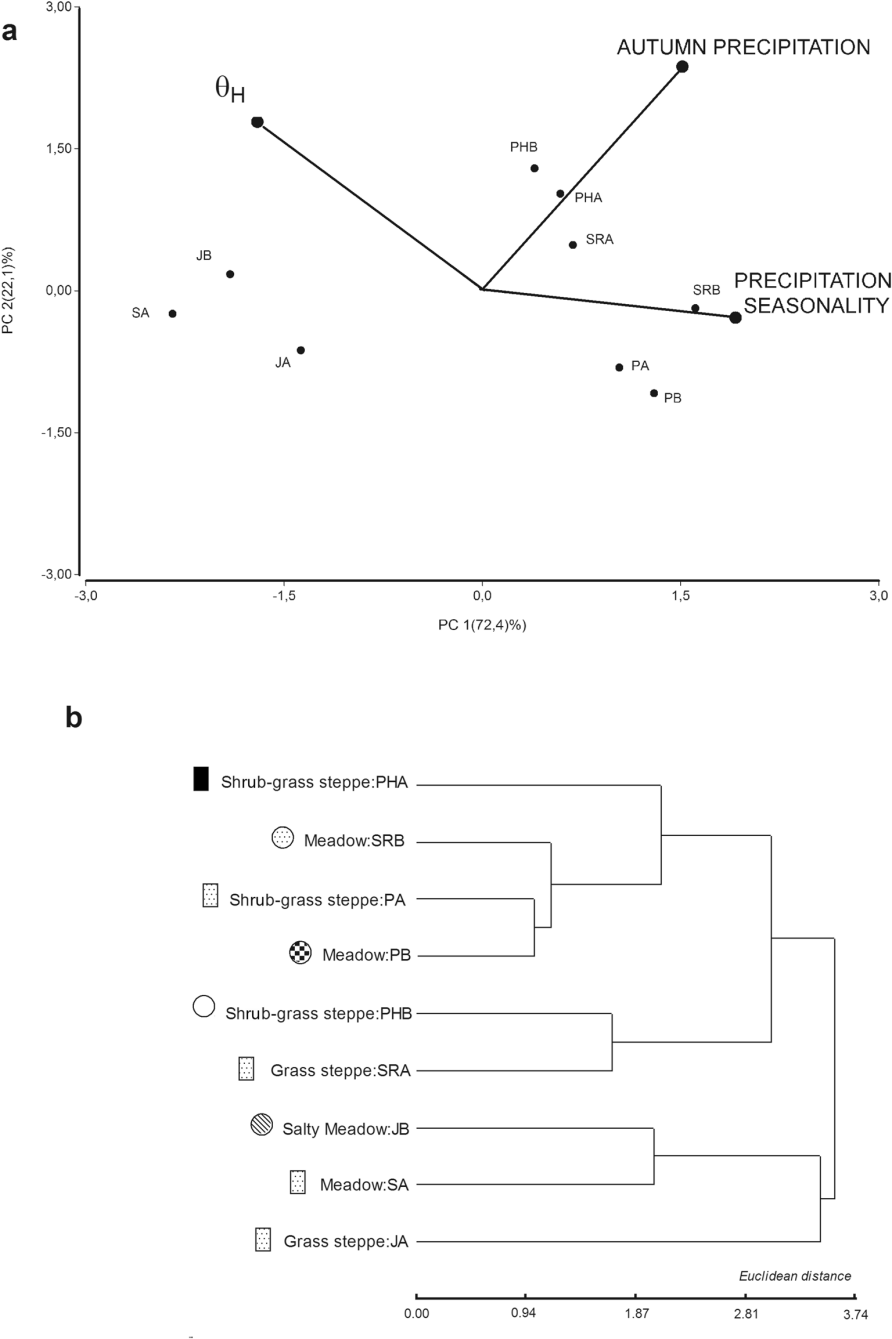
Principal Component Analysis of environmental variables and hydrotime model parameters (**a**) and Dendrogram based on a hierarchical clustering method with environmental and physiognomic characteristics of each sampled site (**b**). Symbols represent the different types of soil as described in Table 1.

The PCA showed that the first two PCs explain 94% of the variation (PC1 = 63%, eigenvalue = 3.16 and PC2 = 31%, eigenvalue = 1.57). The variable with the greatest weight on the first axis was autumn precipitation, and for the second axis, θ_H_ and precipitation seasonality (Fig. 2a). The eastern populations (JA, JB and SA) showed a clear separation from the others. The western populations clustered with autumn precipitation and precipitation seasonality, while the eastern populations (JA, JB and SA) clustered with θ_H_ (Fig. 2a). On the other hand, the hierarchical clustering analyses based on environmental and physiognomic variables showed three groups: one group with eastern populations (JB, SA and JA quite separate from the others), a second group with central-western populations (PA, PB, SRB, PHA) and a third with populations from the Andean and Pre-Andean Mountains (PHB, SRA) (Fig. 2b).

## Discussion

This study quantified the germination response of *Festuca pallescens* seed populations to different levels of water availability by fitting a hydrotime model. This model allowed us to explore differences in the effect of hydric conditions on seed germination, and their association with the adaptive responses of *F. pallescens* populations to their habitats. We found varying patterns of seed response to water availability among populations located along a strong precipitation gradient, with significant differences in the hydrotime (θ_H_), although this variation did not follow the trend of decreasing precipitation. Instead, populations at the extremes of the gradient required the accumulation of more units of MPa days for germination than the central populations. The estimated mean base water potential was relatively low (ψb_(50)_ = − 2.79 ± 0.45 MPa) and did not differ among populations as expected given the very marked variation in annual rainfall. Furthermore, associations with environmental variables were not significant. Altogether, our results indicate that germination responses might be linked to micro-environmental variables at each natural site rather than the landscape-scale gradient, suggesting the existence of local adaptation of pathways involved in the signaling network that underlies the environmental regulation of seed germination. Local adaptations and genetic differences in traits related to germination were proposed for grasses^6,42,43^, annual species^44^ and trees^45,46^.

The ability of seeds to germinate at low water potential is usually associated with adaptation to dry habitats^47–49^. In arid and semiarid environments, however, establishment can fail when seeds germinate after sporadic rain if water is insufficient for subsequent seedling growth (i.e., survival and installation of new individuals)^15^. Therefore, there may be a trade-off between two strategies: “germinating with low water availability” and “germinating after an event/period of abundant rain”. The ψb_(50)_ found in *F. pallescens* was low, but lay within the range of values shown by species of grasses, shrubs and perennial herbs from deserts and semi-deserts^47^. Differences were not significant at an intraspecific level (i.e., between populations from humid and arid sites), suggesting interplay with other factors apart from annual precipitation, such as soil moisture levels during the germination season^48^. Other native species from grass and shrub-grass steppes in North Patagonia, such as *Bromus pictus*, showed low values of ψb (− 1.92 MPa), with significant differences among populations along a latitudinal gradient, but not related to rainfall variation^26^. Moreover, in some locations *F. pallescens* shares environments (i.e., similar climatic conditions) with populations of *Poa ligularis* that show variations in ψb_(50)_ along a latitudinal gradient, from − 0.69 to − 0.84 MPa at 10 °C^6^. These authors found that populations from sites with low mean precipitation (156 mm) during autumn and spring (seasons with cool temperatures) showed lower values of ψb_(50)_ (i.e., more negative). However, these values were still higher (i.e., less negative) than those registered for *F. pallescens* (ψb_(50)_ = − 0.96 vs. ψb_(50)_ = − 2.13 MPa in *P. ligularis* and *F. pallescens,* respectively, for similar precipitation). In addition, perennial grasses in North American (e.g., *Elymus elymoides, Poa secunda*)^47^ and Mediterranean arid ecosystems (e.g., *Stipa tenacissima*^43^ showed ψb_(50)_ values within the range of − 0.73 and − 2.10 MPa). Therefore, *F. pallescens* seeds are able to germinate at low water potentials (between − 2.13 and − 3.14 MPa) and are probably adapted to xeric environments with low water availability, even lower than *P. ligularis*, one of the better adapted or more abundant species in many arid environments without grazing^50–52^. Moreover, our results showed no correlation between seed size and either base water potential or hydrotime for germination, suggesting that adaptation to dry habitats relies on the ability to germinate at low water potential rather than seed size^7^.

Seeds from populations located in optimal environments for *F. pallescens* germinated at all the tested water potentials (e.g., wetlands SRB and PB), while the easternmost populations did not reach full germination at − 1.6 MPa. There are two possible explanations for these results: either these water potentials (− 1 and − 1.6 MPa) are close to the hydric limits that permit seed germination in the species, or the percentage of viable seeds was low. According to the ψb_(50)_ predicted from the hydrotime model, seeds from SA should have germinated easily at the low base water potentials. Future experiments testing the estimated base water potential will confirm whether seeds actually germinate at this hydric potential. Overall, this population had low seed production and viability. By the end of the experiment, most of these seeds had died.

**Figure 3:**
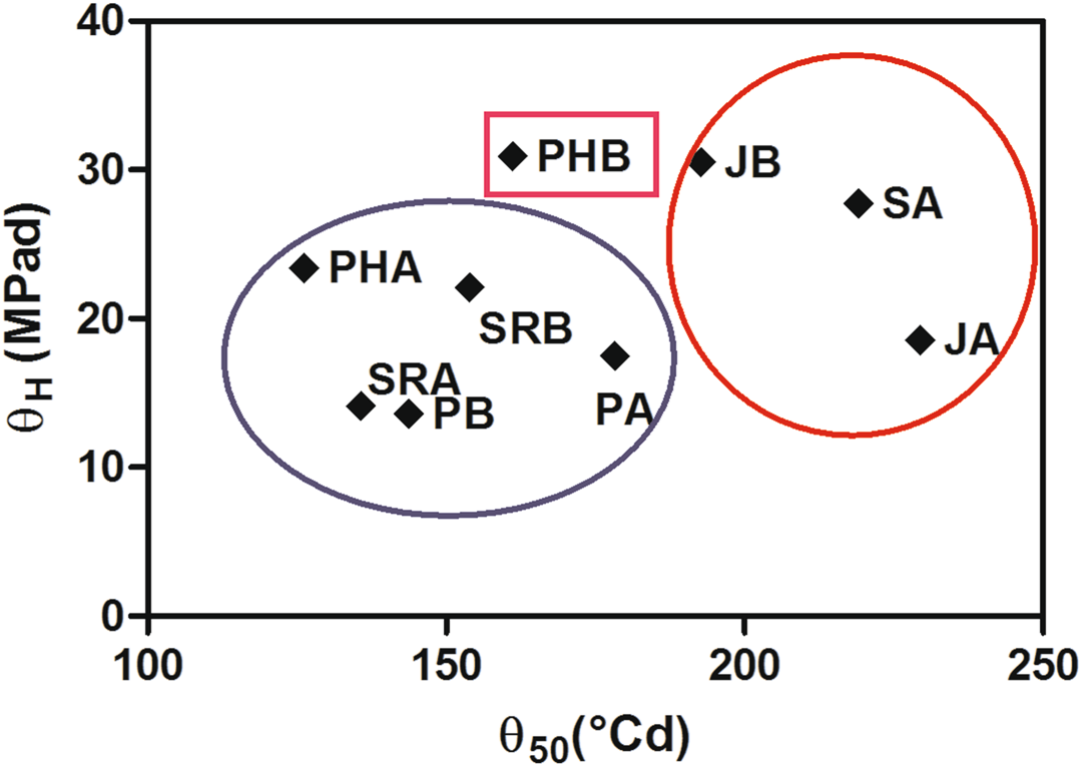
Relation between the thermal time (θ_50_ [°C d) calculated in a previous work^37^) and the hydrotime (θ_H_ [MPa d]), from the present study).

Values for θ_H_ differed between populations, but this difference was not associated with the rainfall gradient, since the highest values of θ_H_ were found in the populations located at both extremes. The σ_ψb_ did not vary significantly among populations, but higher values were associated with higher θ_H_ (e.g., populations JB, SA, SRA, PHB). High θ_H_ values indicate a longer time required for germination, since θ_H_ is related to the period between the start of imbibition and the initiation of radicle emergence (phase II of germination)^12^; in association with high σ_ψb,_ this can result in a greater spread of germination times^19^. In the easternmost sampling sites, annual average rainfall is much lower than in western locations (150–200 mm vs 609–1034 mm). The eastern populations are located in environments with low values of autumn precipitation, low precipitation variability, fairly deep soils and erratic rains. Seeds are therefore likely to germinate only when a prolonged wet period occurs, precluding germination after a short pulse of rain (e.g., during summer dispersion). Autumn germination is thus likely, favoring subsequent seedling survival, as reported for several perennial species^32,37,51,53^. In addition, high values of θ_(50)_ (thermal time) and σ_θ_ (deviation of thermal time)^37^ indicate that the seeds of these populations also require the accumulation of more °C days for germination. Both thermal and hydric conditions are more restrictive for germination in these highly fluctuating environments (Fig. 3). In contrast, populations from the central area of the gradient have fewer germination restrictions, the lowest values of θ_H_ being recorded in PB and SRB, which correspond to optimal environments for *F. pallescens*^30^.

The populations located at higher altitudes in the central-western area, however, showed slightly higher values of θ_H_ (i.e., PHA, PA, SRA). These populations may exhibit more rapid desiccation of the soil surface due to low temperatures and long periods of frost exposure, or fluctuations in water content in the upper soil layers^9^, limiting the water uptake by seeds. Moreover, due to their topographic position on north oriented slopes, populations PHA and SRA are exposed to western winds that erode the soil (i.e., shallow soils) and increase the atmospheric demand for water, producing a negative water balance for species development^54,55^. On the other hand, population PHB unexpectedly shows the highest θ_H_ and the most negative ψb. The environmental setting of this population is very different from the usual fescue sites. It develops in a “steppe-like” environment within a glacial valley that is highly exposed to winds. This community is a shrub-grass steppe dominated by *Mulinum spinosum* and *Pappostipa speciosa* spp, with a low proportion of *F. pallescens*; it grows in sandy-stony soils with a low water-holding capacity^31^. This site corresponds to a cold environment with temperatures that drop below the freezing point during autumn, which has the same effect as water stress for seed populations^56^. In addition, this population was highly differentiated from the others when analyzed with nuclear microsatellites^57^. The taxonomic status was clear, but a putative hybrid origin could not be discarded^58^. The unexpected values recorded for this population may therefore require further research. Overall, micro-environmental cues determined by the complex interaction of meteorological, soil, physical and biological processes may trigger differences in population germination responses^59^.

Many environmental differences exist among the eco-regions where these *F. pallescens* populations were sampled, in particular with respect to precipitation seasonality, autumn precipitation and soil types. However, clustering was not strictly associated with the ecological classification. Whereas the eastern populations were clearly separated (JA, JB and SA), SRB (Pre-Andean ecological region) was similar to PA and PB (Hills and Plateau ecological region). These three populations are also grouped with the rainfall variables (greater precipitation variability and intermediate values of autumn precipitation), suggesting that water availability is higher in PA and PB environments than in the other populations of the Hills and Plateau ecological region. Similarly, in the same nine populations evaluated through a thermal time threshold model^37^, the response of germination to temperature separated the three easternmost populations (JA, JB and SA), which had a significantly higher thermal time and σ_θ_. Thus, the populations inhabiting harsh environments experienced stronger thermal and hydric restrictions for germination.

Altogether, our results suggest that at least along the rainfall gradient evaluated here, when water is a limiting factor (i.e., at the extremes of the rainfall gradient), germination may be prevented or delayed. Moreover, towards the eastern extreme of the rainfall gradient, both thermal-time^37^ and the hydrotime parameters showed high values, indicating that populations from these environments displayed lower germination rates as a result of harsh local conditions. In these environments germination takes place after a prolonged wet period. However, even though western environments might seem more suitable for the species (i.e., more humid and colder, with low evapotranspiration), local environmental characteristics (i.e., soil types) could delay germination in some seed populations. In semi-arid regions, soil organic matter content is low, so these soils tend to become compact when dry, reducing water entry into the soil, for example, after sporadic rains^60^. Towards the west, populations PHA and PHB are situated in extreme humid environments with sandy loam soils, but have different edaphic characteristics (i.e., wind exposure). In the center of the gradient, populations are located in meadows or steppes with fairly deep sandy loam soils. Soils from these environments accumulate more organic matter and volcanic sediments than at the extremes, increasing their capacity for water retention. Most of these populations are thus situated in optimal environments for this species. Towards the east, precipitation is sporadic, soils are less developed, have less organic matter, and their water retention capacity may be lower. Nevertheless, since most soils along the gradient were sandy loam, they probably had similar water retention properties, which would lessen differences between populations in the mean base water potential. In addition, towards the western extreme of the distribution, shrubs and other grass species that dominate these environments compete for resources with *F. pallescens*. This competition could also determine the suitability of certain genotypes for these ecosystems. Combining the hydrotime model results from the present study with information from a previous study that analyzed thermal time^37^, we infer that *F. pallescens* would be able to accumulate thermal time in cold environments (T_b_ = − 0.47°C)^37^, even if soil moisture is relatively low (ψb_(50)_ = − 2.79MPa).

## Conclusion

Germination response to water availability varied among populations of *Festuca pallescens* from different locations along the rainfall gradient that covers their longitudinal distribution range in North Patagonia. The hydrotime (θ_H_) varied among populations, reflecting their germination response to different water potentials. This variation was not clinal, but rather associated with local environments. Micro-environmental cues may therefore influence the physiological behavior of the species regardless of the denoted rainfall gradient. Hence, by combining two approaches we were able to identify differences in the effects of water availability and temperature^37^ on certain processes during germination. These differences suggest the existence of local adaptation that underlies the environmental regulation of seed behavior. A trade-off may occur between temperature and humidity requirements and the "need" to germinate during a relatively prolonged rain event that increases the success of new individuals. Further studies incorporating more populations from the distribution range of this species in Patagonia will increase knowledge of the hydric ranges suitable for their seed germination. These studies will enable the careful selection of seeds that are capable of germination under increasing soil water stress, leading to better outcomes in breeding and restoration programs.

## Supplementary Information

Supplementary Information.
