# Supplementary material for: Germination response to water availability in populations of Festuca pallescens along a Patagonian rainfall gradient based on hydrotime model parameters
Source: Sci Rep. 2021 May 20;11:10653. doi: 10.1038/s41598-021-89901-1 (PMC8137931; doi:10.1038/s41598-021-89901-1)
Supplement: Supplementary file 1 — Supplementary Information. [file 41598_2021_89901_MOESM1_ESM.pdf]

Supplementary Material

Germination response to water availability in populations of *Festuca pallescens* along a Patagonian rainfall gradient based on hydrotime model parameters

López AS, López DR, Arana MV, Batlla D, Marchelli P.

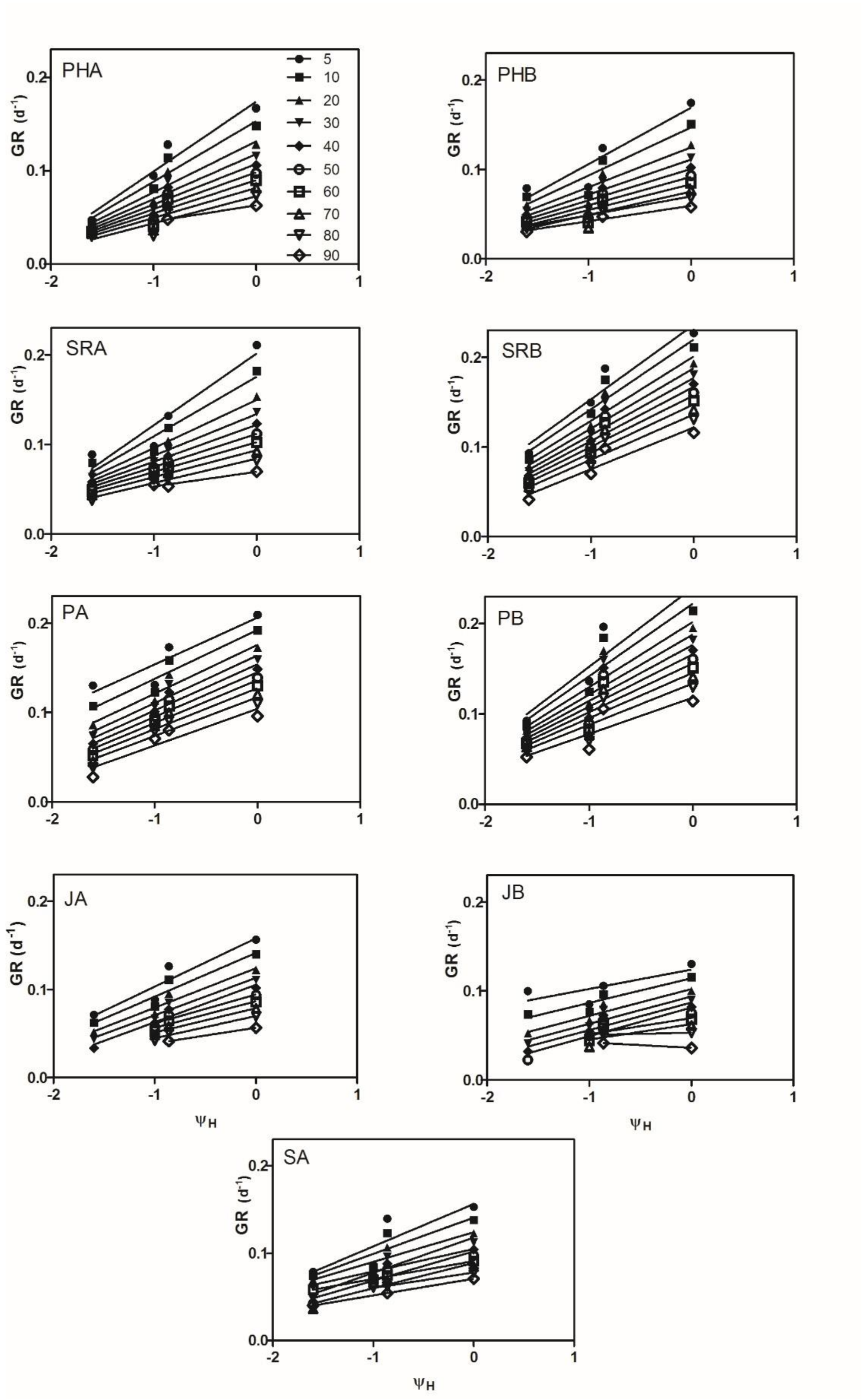

Figure A1: Relationship between the germination rate (GR) of the nine seed populations of *Festuca pallescens* and the water potential. Germination rate for 5 to

90% population fractions were regressed as linear functions of the water potential and intercept the axis of water potentials (GR = 0) in the mean base water potential (ψb) for each population.

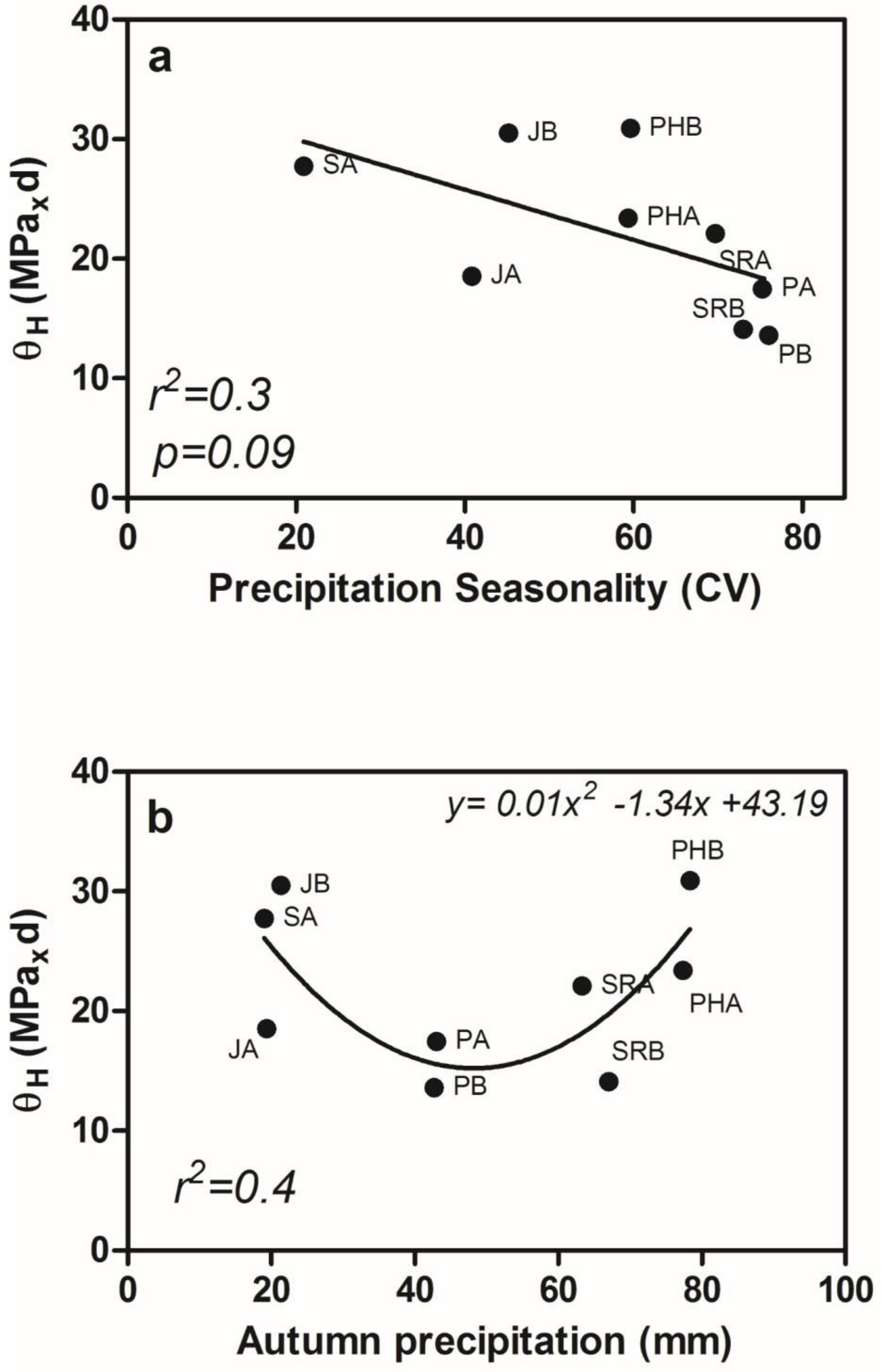

Figure A2: Correlations between hydrotime (θ_H (MPad)) and precipitation seasonality (a) and autumn precipitation (b) in populations of *Festuca pallescens*.

Table A1: Environmental variables and the hydrotime parameters used in the correlations and multivariate analyses.

Population	Precipitation Seasonality	Autumn Precipitation	$\Psi_{b(50)}$ (MPa)	$\sigma_{\Psi b}$	θ_H (MPa/d)	Fisiognomic	Type of soil
PHA	59.34	77.33	-2.59	0.57	23.41	Shrub-grass steppe	Fairly deep volcanic
PHB	59.62	78.33	-3.14	0.91	30.93	Shrub-grass steppe	Deep sandy
SRA	69.68	63.33	-3.05	0.85	22.10	Grass steppe	Fairly deep sandy loam
SRB	72.98	67.00	-2.84	0.60	14.13	Meadow	Deep sandy loam with high proportion of Organic Soil Matter (OSM)
PA	75.25	43.00	-2.91	0.64	17.49	Shrub-grass steppe	Fairly deep sandy loam
PB	76.02	42.66	-2.69	0.58	13.63	Meadow	Deep loamy sand. alkalinic with high proportion of OSM
JA	40.84	19.33	-2.13	0.54	18.55	Grass steppe	Fairly deep sandy loam
JB	45.19	21.33	-2.66	0.79	30.53	Salty Meadow	Deep silty loam. saline-alkaline with high proportion of OSM
SA	20.91	19.00	-3.12	0.74	27.76	Meadow	Fairly deep sandy loam
